# Anatomical Society

**Published:** 1879-04

**Authors:** O. C. Oliver

**Affiliations:** Hotel D’Orient, Paris


### ARTICLE XV.

ANATOMICAL SOCIETY. President, Dr. Charcot.

M. Queneu read a communication on *Lesions of the Anterior Lobes of the Cerebrum.* The patient, a man of 78 years, was brought to the hospital, on the day following an apoplectic attack, in an unconscious condition, with paralysis of the left inferior extremity and superior extremity of the same side, complicated by slight contraction limited to the hand and forearm of the afflicted side. These paralyses persisted until the death of the patient 6 days after of right lobular pneumonia. The autopsy showed upon the external surface of the right hemisphere, at the level of the superior extremity of the ascending parietal convolution, a spot which seemed less firm than the rest of the encephalon. This spot was so superficial at one point that a portion of its contents, a recent, dark colored clot, escaped through a small rent which appeared at the bottom of the fissure, separating the paracentral from the square lobe. Sections vertical and parallel to the "sulcus of Ronaldo," revealed a hæmorrhagic clot occupying a very small part of the *superior frontal* fasces, the greater part of the superior parietal fasces and rather more than half the superior pediculo-parietal fasces. All the neighboring parts, the corpus striatum, etc., were absolutely unchanged. The great interest of this observation is the rarity of such a lesion occurring in this region, so limited in its implication of the structures adjacent to its seat. The speaker mentioned a case communicated by M. Pitres. The patient, a woman of 65 years, became hemiplegic from an apoplectic attack in 1873. Six months afterwards the patient had an epileptic fit, acting upon the sound (left) side. These attacks repeated themselves in '74, '75 and '76, when the patient died. The autopsy revealed an ochreous clot of the size of an almond, extending from the base of the first right frontal convolution to the base of superior parietal lobule, and extending beneath as far as the superior extremities of the two ascending convolutions. The speaker also gave the notes of two cases in the service of Prof. Hardy. The first patient, a man of 65 years, was brought to the hospital with complete hemiplegia of the right side following an apoplectic attack. At the end of about 7 weeks contraction and atrophy of the paralyzed members supervened. Despite every precaution, a large "bed sore" formed, with diffuse phlegmonous inflammation with septicæmia, to which the patient succumbed. The autopsy revealed a hæmorrhagic clot situated in the superior fronto-parietal fasces of the left centrum ovale. The second case was that of an old man of 88 years, brought to La Charité Hospital unconscious from an apoplectic stroke, and which presented, as a unique symptom, paralysis limited to the right members, which at the end of 8 days limited itself to the right superior member, persisting so without amendment or contraction till the death of the patient 7 weeks after. The autopsy revealed, besides atheromatous degeneration of the aorta and cerebral vessels, a spot of softening in the left hemisphere, which had the form of a broad fissure extending in an oblique direction as regards the fissure separating the two hemispheres, its anterior extremity situated at the summit of the cone formed by the inferior parietal fasces, its posterior at the sphenoidal fasces. M. Queneu then says that in these cases the lesions were so definitely limited as to render the clinical phenomena as clearly intelligible and as important diagnostically in locating lesions of the cerebrum as physiological experiments. M. Landouzy wished to suggest that the anomalous symptom presented by the patient, namely, contracture of the muscles of the hand and forearm, might be due to the irritation of the gray matter of the brain, since it was shown to have been lacerated at one point by the clot. The speaker had observed a case of hemiplegia (?) following apoplexy, in which case there were contractures of the hand and forearm, which appeared to be due to excitation of the cortical substance superficially intermediate to the paracentral and square lobes. M. Queneu said, in contrast to these cases with well marked symptoms, he wished to mention one in which every known rule of cerebral pathology seemed to have been violated, so to speak. This case was that of a tuberculous patient of 43 years, in whose brain he found a tuberculous deposit as large as a pea, in the inferior fronto-parietal fasces, and yet this patient had never presented a single symptom of cerebral trouble.

O. C. OLIVER.

HOTEL D'ORIENT, PARIS, February 28, 1879.

